# PD-L1 expression and mismatch repair deficiency in locally advanced head and neck squamous cell carcinoma treated with chemoradiotherapy: association with treatment response and survival

**DOI:** 10.3389/fimmu.2025.1709512

**Published:** 2026-01-12

**Authors:** Bingyi Zhang, Dong Guo, Yujuan Hou, Xinyue Wei, Yuanyuan Cai, Daqing Sun, Xiaoxiao Wang, Xiaoli Wang, Jianwen Li, Furong Hao

**Affiliations:** Department of Radiation Oncology, Weifang People’s Hospital, Shandong Second Medical University, Weifang, Shandong, China

**Keywords:** biomarker, chemoradiotherapy, dMMR, locally advanced head and neck squamous cell carcinoma, PD-L1

## Abstract

**Background:**

Concurrent chemoradiotherapy (CCRT) served as the cornerstone of definitive treatment for locally advanced head and neck squamous cell carcinoma (LA-HNSCC), yet there was heterogeneity in patients’ treatment responses. This study aimed to evaluate the clinical significance of programmed death ligand-1 (PD-L1) expression and deficient mismatch repair (dMMR) as predictive markers of response to CCRT in patients with LA-HNSCC.

**Methods:**

This study retrospectively analyzed 156 cases of LA-HNSCC patients who received CCRT treatment at our hospital from January 2020 to April 2024. All patients received intensity-modulated radiotherapy with concurrent chemotherapy. PD-L1 expression was assessed using immunohistochemistry and quantified with the Combined Positive Score (CPS). Mismatch repair status was evaluated using antibodies against MLH1, PMS2, MSH2, and MSH6. The treatment response was assessed according to the Response Evaluation Criteria In Solid Tumors version 1.1 (RECIST 1.1).

**Results:**

This study included a total of 156 patients with LA-HNSCC, with a male predominance (125 cases). The deficient mismatch repair (dMMR) prevalence was 6.4% (10/156), and high PD-L1 expression (CPS≥20) was observed in 47.4% (74/156) of patients. There was a significant association between high PD-L1 expression and an increased objective response rate to CCRT (97.1% compared to 58.5%, P<0.001). Conversely, patients with dMMR exhibited a reduced response rate (50.0% versus 79.5%, P = 0.031). In the survival analysis, patients with high PD-L1 expression demonstrated significantly longer overall survival (OS) (P = 0.0021) and progression-free survival (PFS) (P<0.001). In contrast, dMMR status was not significantly associated with OS or PFS. Multivariate analysis identified smoking (HR = 6.871, 95%CI: 2.214-21.32, P<0.001) and high PD-L1 expression (HR = 2.591, 95%CI: 1.036-6.483, P = 0.042) as independent risk factors for OS.

**Conclusion:**

The study confirmed that high PD-L1 expression was a positive biomarker for predicting the benefit of CCRT in patients with LA-HNSCC. Although dMMR was associated with initial treatment resistance, its unique immunogenicity was found to influence disease progression and subsequent treatment options. These findings suggested that MMR testing in LA-HNSCC patients had significant clinical value, not only aiding in the identification of patients who might benefit from subsequent immunotherapy but also potentially informing initial treatment strategies, such as the prospective exploration of combined immunotherapy approaches.

## Introduction

Head and neck squamous cell carcinoma (HNSCC) ranks among the top ten most prevalent cancers worldwide, with its incidence in males typically ranging between the sixth and eighth positions (1). GLOBOCAN 2022 data indicate that HNSCC, comprising malignancies of the oral cavity, oropharynx, and larynx, is responsible for approximately 930,000 new incidences and 467,000 mortalities annually on a global scale (2). According to data released by the National Cancer Center of China, the incidence of HNSCC in China has demonstrated an increasing trend, driven by population aging and lifestyle changes (3). This increase is especially significant in high-risk groups, including individuals who use tobacco, consume alcohol, and have human papillomavirus (HPV) infections (4). Because early symptoms are relatively subtle, approximately 60%-70% of patients with HNSCC were found to be in locally advanced stages at their initial medical consultation (5). Due to the tumor’s large size, invasion into adjacent tissues, and frequent involvement of regional lymph nodes, the prognosis for locally advanced HNSCC (LA-HNSCC) is often challenging. The 5-year survival rate for these patients is typically around 30%-50% (6). The main treatment approach for LA-HNSCC was concurrent chemoradiotherapy (CCRT). Despite multimodal treatments including surgery, chemoradiotherapy, and Immune checkpoint blockade (ICB), prognosis for locally advanced HNSCC remains poor. We urgently need to identify predictive biomarkers to better forecast the effectiveness of chemoradiotherapy and provide personalized treatment. Currently, apart from the clear prognostic value of p16 positivity in oropharyngeal squamous cell carcinoma, other predictive factors remain ambiguous (7). Previous studies have confirmed that programmed death-ligand 1 (PD-L1) and deficient mismatch repair (dMMR) are crucial in the pathogenesis and prognostic prediction of malignant tumors, making them focal points in current research (8, 9).

Tumor immunosuppression and immune evasion are central mechanisms enabling cancers to escape immune destruction, with the PD-1 (Programmed Death-1)/PD-L1 pathway serving as a critical immune checkpoint (10–12). Binding of PD-L1 on tumor cells to PD-1 on T cells recruits SHP2, leading to dephosphorylation of T cell receptor signaling molecules and inhibition of T-cell activation (13, 14). This suppression facilitates tumor immune evasion. Conversely, dMMR, which involves loss of function in DNA mismatch repair (MMR) genes such as MLH1, MSH2, MSH6, or PMS2, leads to high microsatellite instability (MSI-H) and increased tumor mutational burden Conversely, dMMR involves the loss of function in DNA mismatch repair genes, including MLH1, MSH2, MSH6, or PMS2, which in turn leads to increased microsatellite instability (MSI-H) and a rise in tumor mutational burden (15, 16). This genomic instability generates abundant neoantigens that promote antitumor immunity through enhanced antigen presentation and T-cell infiltration, thereby creating an immunogenic “hot tumor” microenvironment—characterized by robust CD8^+^ T-cell infiltration and frequently elevated PD-L1 expression (17, 18). ICB with PD-1/PD-L1 inhibitors counteracts immunosuppression by restoring T-cell function, showing expanding clinical utility in HNSCC (19, 20), while dMMR/MSI-H tumors exhibit heightened sensitivity to ICIs due to their enhanced immunogenicity (21–23). However, the predictive value of PD-L1 and dMMR for the efficacy of chemoradiotherapy in LA-HNSCC remains unclear.

This study aimed to systematically evaluate the predictive value of dMMR and PD-L1 expression for the efficacy of chemoradiotherapy in patients with LA-HNSCC. By analyzing clinical data and pathological information, we aim to assess whether these biomarkers can serve as effective indicators of treatment response and survival outcomes.

## Materials and methods

### Patient selection

We retrospectively collected 156 patients with LA-HNSCC who underwent concurrent chemoradiotherapy at Weifang People’s Hospital between January 2020 and April 2024. This study was approved by the ethics review committee of Weifang People’s Hospital (Approval Number: KYLL20241008-18). The requirement for informed consent was waived because the data were anonymized. All procedures were conducted in accordance with relevant guidelines and regulations. Eligibility criteria comprised: (1) Staging as T3-4/N+ per the American Joint Committee on Cancer (AJCC) 8th edition; (2) Histopathological confirmation of squamous cell carcinoma via biopsy specimen; (3) Absence of prior head and neck radiotherapy, history of other malignancies, or significant comorbidities. Exclusion criteria included: (1) Incomplete radiotherapy; (2) Pregnancy or lactation; (3) Non-squamous cell carcinoma histology; (4) Incomplete clinical data or insufficient pathological tissue.

### Treatment Regimen

All patients received concurrent chemoradiotherapy. Although surgical resection is a standard option for oral cavity tumors and may be considered in younger patients (≤70 years), the decision to pursue definitive CCRT in this cohort was based on one or more of the following factors: patient preference for organ preservation, medical contraindications to surgery (cardiopulmonary comorbidities), locally advanced disease with anticipated poor functional outcomes post-resection (T4 disease involving bone or deep musculature), or multidisciplinary tumor board recommendation favoring non-surgical approach to preserve swallowing and speech function. Radiotherapy was delivered using intensity-modulated radiotherapy (IMRT). The prescribed dose to the primary tumor and metastatic cervical lymph nodes was 66.0-70.0 Gy. The high-risk clinical target volume (CTV-H), including lymph node drainage areas, is set at 60.0 Gy, while the low-risk volume (CTV-L) is set at 50.0-54.0 Gy. Conventional fractionated radiotherapy was administered at 1.8-2.0 Gy per session, five times a week. Concurrent chemotherapy consisted of cisplatin (80–100 mg/m²) or nedaplatin (80–100 mg/m²), administered typically every 3 weeks. The number of cycles was determined by patient tolerance and treatment protocol.

### Immunohistochemistry of PD-L1 and dMMR

All enrolled cases underwent standardized immunohistochemical (IHC) staining. MMR status was assessed using monoclonal antibodies (Anti-MLH1, PMS2, MSH2, MSH6); loss of nuclear staining in tumor cells for any protein dMMR. PD-L1 expression was evaluated using the 22C3 pharmDx assay (Dako) and reported as Combined Positive Score (CPS=PD-L1-stained cells (tumor cells + immune cells)/viable tumor cells×100). Epidermal growth factor receptor (EGFR) membrane expression was assessed; >10% positive tumor membrane staining defined overexpression. p16 protein served as a surrogate marker for HPV infection; diffuse strong nuclear/cytoplasmic staining in ≥70% tumor cells defined positivity. All sections were fixed in 10% neutral buffered formalin (time from resection to fixation ≤ 30 minutes), embedded in paraffin (4 μm thickness), and independently evaluated by two experienced pathologists.

### The efficacy of chemoradiotherapy was assessed according to RECIST 1.1

Treatment response was evaluated using Response Evaluation Criteria in Solid Tumours version 1.1 (RECIST 1.1). Baseline contrast-enhanced CT or MRI was used to define target lesions, with the sum of their diameters calculated; all other lesions were designated as non-target lesions. Imaging reassessment was performed every 2 cycles. Final efficacy assessment was performed 3 months after the completion of radiotherapy. Imaging evaluation was conducted independently by two radiologists from our institution, each with over five years of experience in head and neck tumor imaging. Reviews were performed using the same GE Advantage Workstation (AW) software, version 4.7. Standardized window settings were applied for interpretation: soft tissue windows (width: 350–400 HU; level: 40–60 HU) and bone windows (width: 2000–4000 HU; level: 400–600 HU). Complete response (CR) was defined as the disappearance of all target lesions, resolution of non-target lesions, and no new lesions. Partial response (PR) required a ≥30% decrease in the sum of target lesion diameters from baseline, non-progression of non-target lesions, and no new lesions. Stable disease (SD) indicated target lesion changes that did not meet the thresholds for PR or progressive disease (PD), non-progression of non-target lesions, and no new lesions. PD was defined as a ≥20% increase in the sum of target lesion diameters from the nadir, unequivocal progression of non-target lesions, or the appearance of new lesions.

### Follow-up

Following treatment completion, patients entered a standardized follow-up protocol: quarterly for the first 2 years, semi-annually during years 3-5, and annually thereafter, until June 2025. The evaluation comprises specialized head and neck examinations (including fiberoptic nasopharyngolaryngoscopy), neck ultrasound (semi-annual) with optional contrast-enhanced CT/MRI (annual or semi-annual for high-risk patients), chest CT scans, and laboratory tests including hematological, hepatic/renal function, thyroid function, and tumor markers such as squamous cell carcinoma antigen (SCC) and carcinoembryonic antigen (CEA). Overall survival (OS) and progression-free survival (PFS) were recorded. OS was determined from the treatment commencement date until death or the last recorded follow-up, whichever occurred first. PFS was calculated from the treatment start date to the date of disease progression, death, or the final follow-up, whichever occurred first.

### Statistical analysis

All data were processed using GraphPad Prism 10.4.1 and R 4.5.0. Continuous data are summarized as mean ± standard deviation (x ± s) or median (interquartile range), while categorical variables are reported as frequency (%). Between-group analyses involving small sample sizes or low expected frequencies were compared using Fisher’s exact test. Overall (OS) and progression-free (PFS) survival were analyzed by the Kaplan-Meier method, with group comparisons made using the log-rank test. Multivariate Cox proportional hazards models were employed to calculate hazard ratios (HR) and 95% confidence intervals. Variables with a P-value < 0.05 in the univariate analysis were included in the multivariate models.

## Results

### Baseline characteristics of patients

**Figure 1:**
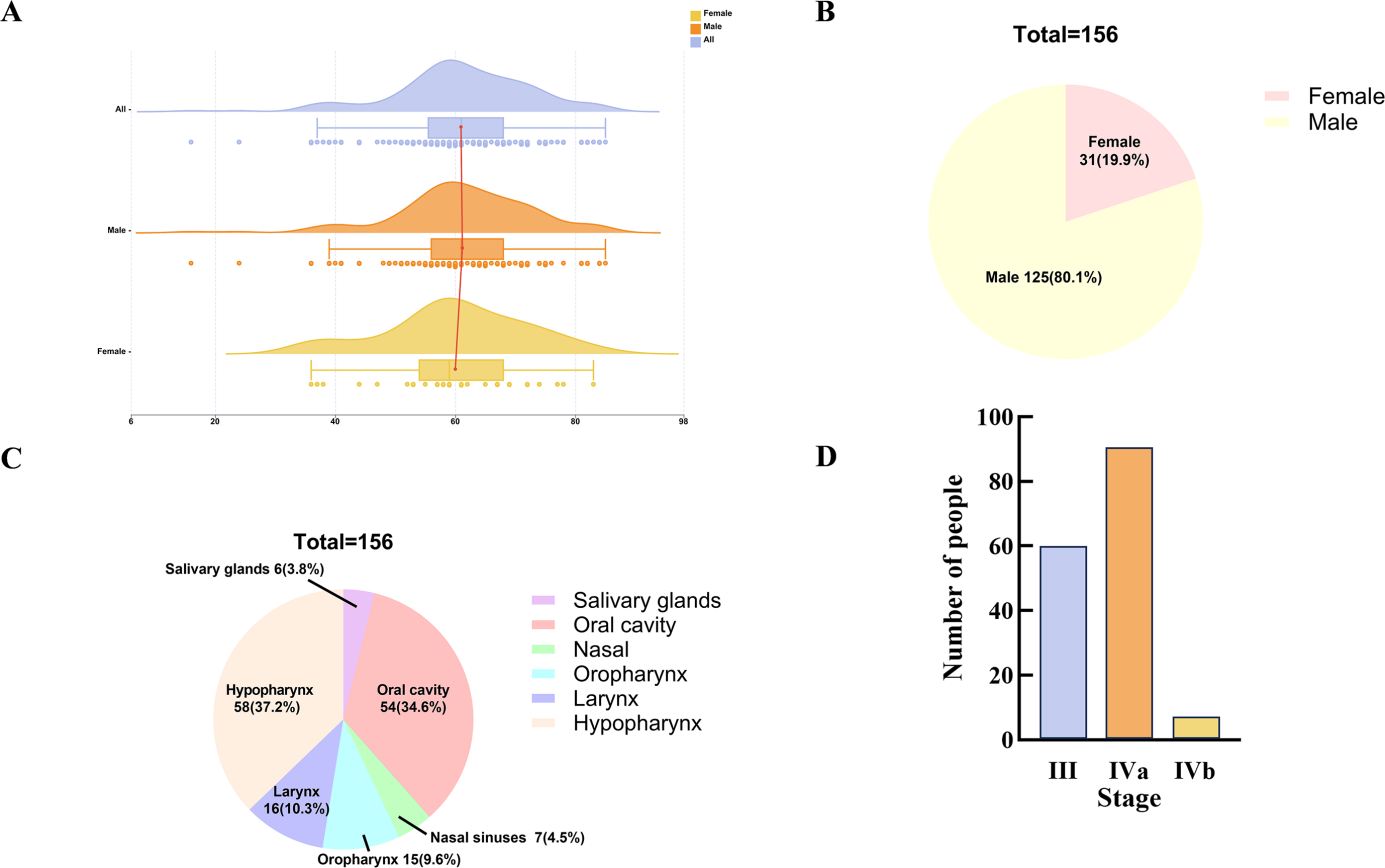
Age distribution and baseline characteristics of 156 cases of LA-HNSCC. **(A)** Age distribution, **(B)** Gender ratio, **(C)** Tumor site, **(D)** Clinical stage. LA-HNSCC, locally advanced head and neck squamous cell carcinoma.

This study enrolled 156 patients with LA-HNSCC, comprising 125 males and 31 females (**Figure 1A**). The median age at diagnosis was 61 years (**Figure 1B**). The median follow-up was 30.6 months. **Figure 1C** depicts the primary tumor sites among all enrolled patients, with the majority occurring in the hypopharynx (n=58, 37.2%), oral cavity (n=54, 34.6%), oropharynx (n=15, 9.6%), salivary glands (n=6, 3.8%), and nasal cavity/paranasal sinuses (n=7, 4.5%). According to the AJCC 8th edition staging system, 59 patients (37.8%) were classified as stage III, 90 patients (57.7%) as stage IVa, and 7 patients (4.5%) as stage IVb, with the distribution detailed in **Figure 1D**.

### dMMR/MSI status and PD-L1 expression

**Figure 2:**
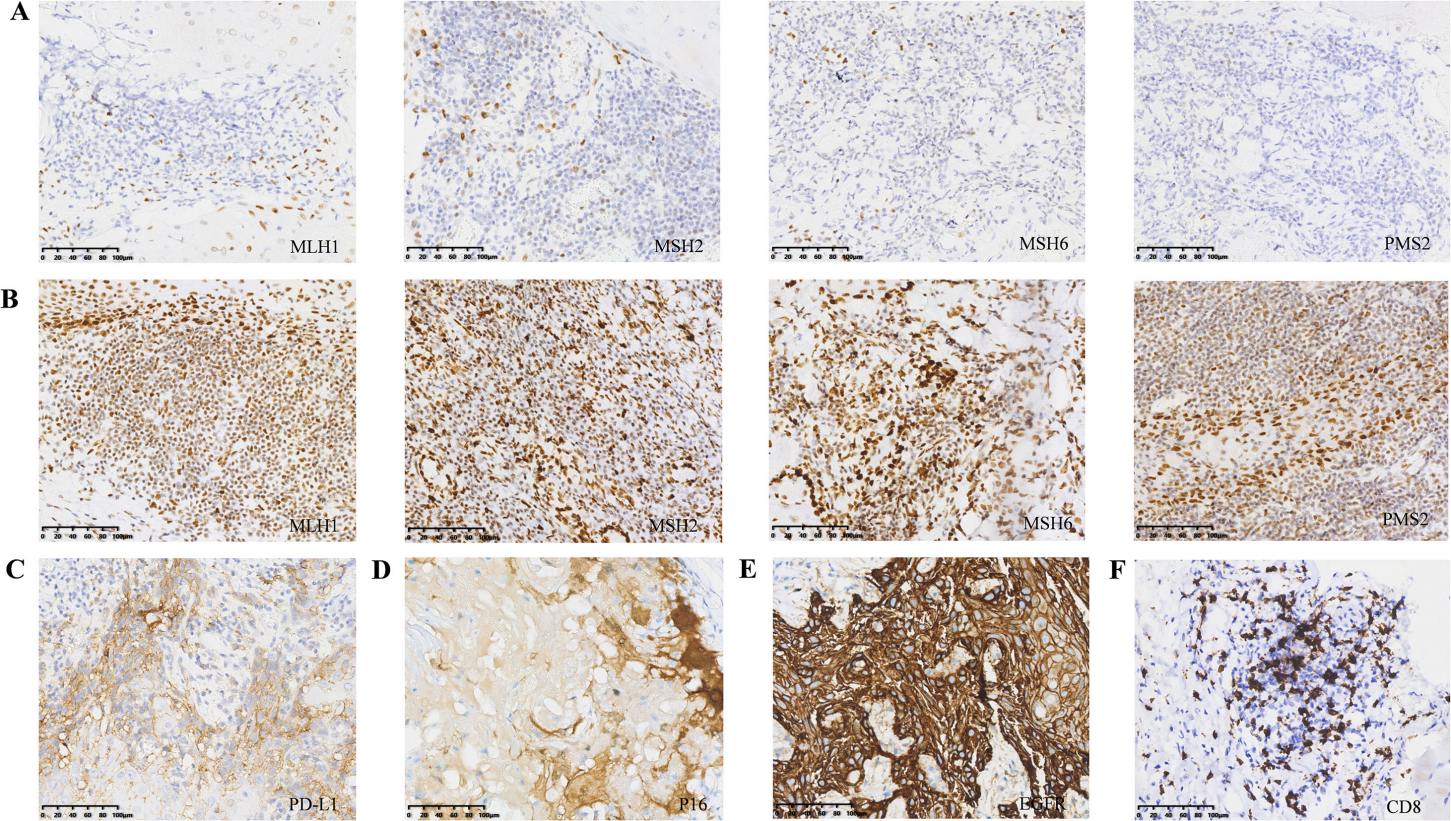
Immunohistochemistry in a subset of patients. **(A)** Loss of MLH1, PMS2, MSH2, MSH6 proteins in tumor cells; **(B)** Intact expression of MLH1, PMS2, MSH2, MSH6 proteins in tumor cells; **(C)** Diffuse and strong membranous expression of PD-L1 in tumor cells; **(D)** Diffuse nuclear and cytoplasmic expression of p16; **(E)** Overexpression of EGFR protein; **(F)** CD8 positive TILs. LA-HNSCC, locally advanced head and neck squamous cell carcinoma; PD-L1, programmed death ligand-1; TILs, Tumor-Infiltrating Lymphocytes.

Immunohistochemical analysis revealed that, among the 156 patients, 10 exhibited losses of MMR protein expression (**Figure 2A**), while the remaining 146 patients displayed intact expression of all four MMR proteins (**Figure 2B**). Of the 10 patients with dMMR, 4 patients exhibited isolated loss of PSM2, 5 patients showed concurrent loss of MLH1 and PSM2, and 1 patient demonstrated complete loss of all four MMR proteins (**Table 1**). A total of 74 patients (47.4%) exhibited PD-L1 expression with a CPS ≥ 20, while 63 patients (40.3%) had CPS values between 1 and 20, and 20 patients (12.8%) had CPS values less than 1 (**Figure 2C**). Overexpression of p16 protein was detected in 17 patients (10.9%) (**Figure 2D**). EGFR mutations were detected in 137 patients (87.8%) (**Figure 2E**), and high CD8^+^ infiltration was observed in 49 patients (31.4%) (**Figure 2F**).

**Table 1:** Distribution of MMR protein expression status in 156 LA-HNSCC cases.

MMR status	MLH1	MSH2	MSH6	PMS2	N
pMMR	+	+	+	+	146 (93.6%)
dMMR	–	–	–	–	2 (1.3%)
dMMR	–	+	+	–	3 (1.9%)
dMMR	+	–	+	+	2 (1.3%)
dMMR	+	+	+	–	3 (1.9%)

pMMR, Proficient Mismatch Repair; dMMR, Deficient Mismatch Repair.

### MMR/PD-L1 molecular subtype classification and its correlation with clinicopathological features

The results of the correlation analysis between MMR and PD-L1 expression and clinicopathological features were shown in **Tables 2**, **3**. Statistical analysis revealed no significant differences in most clinicopathological features (such as age, gender, smoking, alcohol consumption, T/N stage, overall stage, grade, PD-L1, P16, targeted therapy, and immunotherapy) between the pMMR and dMMR groups (all P values>0.05). The P value for EGFR was 0.075, suggesting a potentially higher proportion of EGFR negativity in the dMMR group (30.0% vs 11.0%), although this was not statistically significant (commonly using P<0.05 as the threshold). However, PD-L1 high expression was statistically significantly associated with non-smoking, oral cavity primary, early T stage, advanced N stage, and P16 positivity. PD-L1 expression did not show statistically significant differences across various patient groups categorized by age, gender, history of alcohol consumption, TNM stage, tumor differentiation, MMR status, CD8-positive T cell infiltration, EGFR status, and prior history of targeted/immunotherapy (all P>0.05).

**Table 2:** Baseline patient characteristics stratified by PD-L1 expression status.

Characteristic	N	PD-L1		P value
		CPS>20	CPS ≤ 20	
Total	156	74 (47.4%)	82 (52.6%)	
Age				0.892
≤70	123 (78.8%)	58 (78.4%)	65 (79.3%)	
>70	33 (21.2%)	16 (21.6%)	17 (20.7%)	
Gender				0.356
Female	31 (19.9%)	17 (23.0%)	14 (17.1%)	
Male	125 (80.1%)	57 (77.0%)	68 (82.9%)	
Smoking history				0.004
No	74 (47.4%)	44 (59.5%)	30 (36.6%)	
Yes	82 (52.6%)	30 (40.5%)	52 (63.4%)	
Alcohol history				0.593
No	64 (41.0%)	32 (43.2%)	32 (39.0%)	
Yes	92 (59.0%)	42 (56.8%)	50 (61.0%)	
Tumor site				0.027
Salivary glands	6 (3.8%)	2 (2.7%)	4 (4.9%)	
Oral cavity	54 (34.6%)	33 (44.6%)	21 (25.6%)	
Nasal sinuses	7 (4.5%)	5 (6.8%)	2 (2.4%)	
Oropharynx	15 (9.6%)	9 (12.2%)	6 (7.3%)	
Larynx	16 (10.3%)	5 (6.8%)	11 (10.3%)	
Hypopharynx	58 (37.2%)	20 (37.2%)	38 (46.3%)	
T-stage				0.031
T1-2	58 (37.2%)	34 (45.9%)	24 (29.3%)	
T3-4	98 (62.8%)	40 (54.1%)	58 (70.7%)	
N-stage				0.004
N0-1	80 (51.3%)	29 (39.2%)	51 (62.2%)	
N2-3	76 (48.7%)	45 (60.8%)	31 (37.8%)	
Stage				0.514
III	57 (36.5%)	29 (39.2%)	28 (34.1%)	
IV	99 (63.5%)	45 (60.8%)	54 (65.9%)	
Grade				0.291
Well/Moderate	114 (73.1%)	57 (77.0%)	57 (69.5%)	
Poor/Undifferentiated	42 (26.9%)	17 (23.0%)	25 (30.5%)	
MMR				0.626
pMMR	146 (93.6%)	70 (95.6%)	76 (92.7%)	
dMMR	10 (6.4%)	4 (5.4%)	6 (7.3%)	
CD8				0.794
High	49 (31.4%)	24 (32.4%)	25 (30.5%)	
Low	107 (68.6%)	50 (67.6%)	57 (69.5%)	
EGFR				0.628
+	137 (87.8%)	64 (86.5%)	73 (89.0%)	
-	19 (12.2%)	10 (13.5%)	9 (11.0%)	
P16				0.043
+	17 (10.9%)	12 (16.2%)	5 (6.1%)	
-	139 (89.1%)	62 (83.8%)	77 (93.9%)	
Targeted therapy				0.427
No	104 (66.7%)	47 (63.5%)	57 (69.5%)	
Yes	52 (33.3%)	27 (36.5%)	25 (30.5%)	
Immunotherapy				0.853
No	144 (92.3%)	68 (91.9%)	76 (92.7%)	
Yes	12 (7.7%)	6 (8.1%)	6 (7.3%)	

MMR, Mismatch Repair; pMMR, Proficient Mismatch Repair; dMMR, Deficient Mismatch Repair; EGFR, Epidermal Growth Factor Receptor.

**Table 3:** Baseline patient characteristics stratified by MMR status.

Characteristic	N	MMR		P value
		pMMR	dMMR	
Total	156	146 (93.6%)	10 (6.4%)	
Age				0.131
≤70	123 (78.8%)	117 (80.1%)	6 (60.0%)	
>70	33 (21.2%)	29 (19.9%)	4 (40.0%)	
Gender				0.992
Female	31 (19.9%)	29 (19.9%)	2 (20.0%)	
Male	125 (80.1%)	117 (80.1%)	8 (80.0%)	
Smoking_history				0.254
No	74 (47.4%)	71 (48.6%)	3 (30.0%)	
Yes	82 (52.6%)	75 (51.4%)	7 (70.0%)	
Alcohol_history				0.551
No	64 (41.0%)	59 (40.4%)	5 (50.0%)	
Yes	92 (59.0%)	87 (59.6%)	5 (50.0%)	
T_stage				0.245
T1-2	58 (37.2%)	56 (38.4%)	2 (20.0%)	
T3-4	98 (62.8%)	90 (61.6%)	8 (80.0%)	
N_stage				0.569
N0-1	80 (51.3%)	74 (50.7%)	6 (60.0%)	
N2-3	76 (48.7%)	72 (49.3%)	4 (40.0%)	
Stage				0.814
III	57 (36.5%)	53 (36.3%)	4 (40.0%)	
IV	99 (63.5%)	93 (63.7%)	6 (60.0%)	
Grade				0.610
Well/Moderate	114 (73.1%)	106 (72.6%)	8 (80.0%)	
Poor/Undifferentiated	42 (26.9%)	40 (27.4)	2 (20.0%)	
PD-L1				0.626
CPS≥20	74 (47.4%)	70 (47.9%)	4 (40.0%)	
CPS<20	82 (52.6%)	76 (52.1%)	6 (60.0%)	
EGFR				0.075
+	137 (87.8%)	130 (89.0%)	7 (70.0%)	
-	19 (12.2%)	16 (11.0%)	3 (30.0%)	
P16				0.925
+	17 (10.9%)	16 (11.0%)	1 (10.0%)	
-	139 (89.1%)	130 (89.0%)	9 (90.0%)	
Targeted therapy				0.817
No	104 (66.7%)	97 (66.4%	7 (70.0%)	
Yes	52 (33.3%)	49 (33.6%)	3 (30.0%)	
Immunotherapy				0.131
No	144 (92.3%)	136 (93.2%)	8 (80.0%)	
Yes	12 (7.7%)	10 (6.8%)	2 (20.0%)	

MMR, Mismatch Repair; pMMR, Proficient Mismatch Repair; dMMR, Deficient Mismatch Repair; EGFR, Epidermal Growth Factor Receptor.

**Figure 3:**
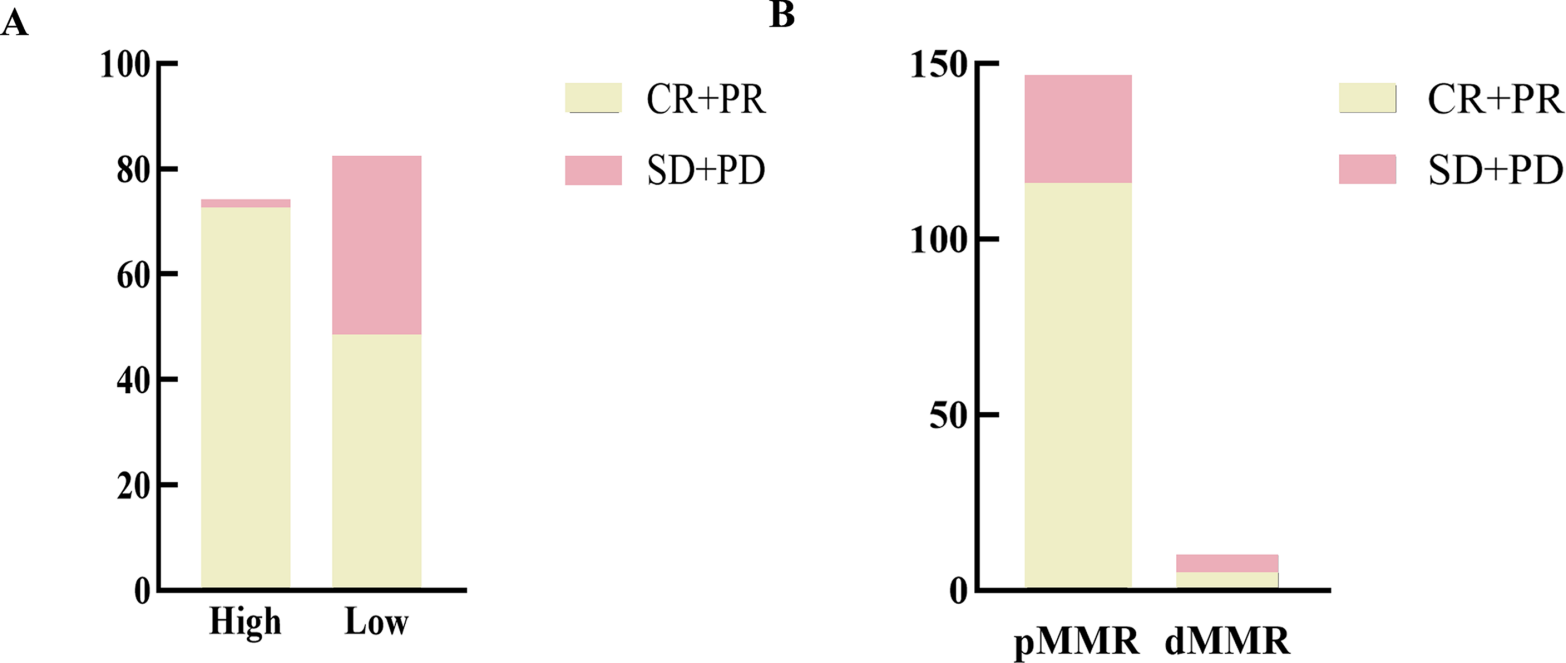
Relationship between PD-L1 expression or MMR status and clinical response to CCRT. **(A)** Treatment response in LA-HNSCC patients with low or high PD-L1 expression. Patients with high PD-L1 expression had a higher response rate to CCRT (P<0.001). **(B)** Treatment response in LA-HNSCC patients with pMMR or dMMR. Patients with dMMR had a lower response rate to CCRT (P = 0.031). CR, complete response; PR, partial response; SD, stable disease; PD, progressive disease.

A total of 83 patients achieved CR, 39 patients achieved PR, 31 patients showed SD, and 4 patients developed PD (**Figure 3**). Data analysis revealed a significantly higher response rate in the high PD-L1 expression group (97.1%) compared to the low expression group (58.5%) (P<0.001). The dMMR status was associated with poorer CCRT efficacy. The treatment effectiveness rate of the pMMR group (79.5%) was higher than that of the dMMR group (50.0%), which is statistically significant (P = 0.031) (**Table 4**).

**Table 4:** The relationship between PD-L1 expression or MMR status and clinical response to CCRT.

Biomarker	Subgroup	Treatment response		P value
		CR/PR	SD/PD	
PD-L1	CPS>20	73 (97.1%)	1 (2.9%)	<0.001
	CPS ≤ 20	48 (58.5%)	34 (41.5%)	
MMR	pMMR	116 (79.5%)	30 (20.5%)	0.031
	dMMR	5 (50.0%)	5 (50.0%)	
Total		121 (77.6%)	35 (22.4%)	

MMR, Mismatch Repair; pMMR, Proficient Mismatch Repair; dMMR, Deficient Mismatch Repair; EGFR, Epidermal Growth Factor Receptor.

### Survival analysis

**Figure 4:**
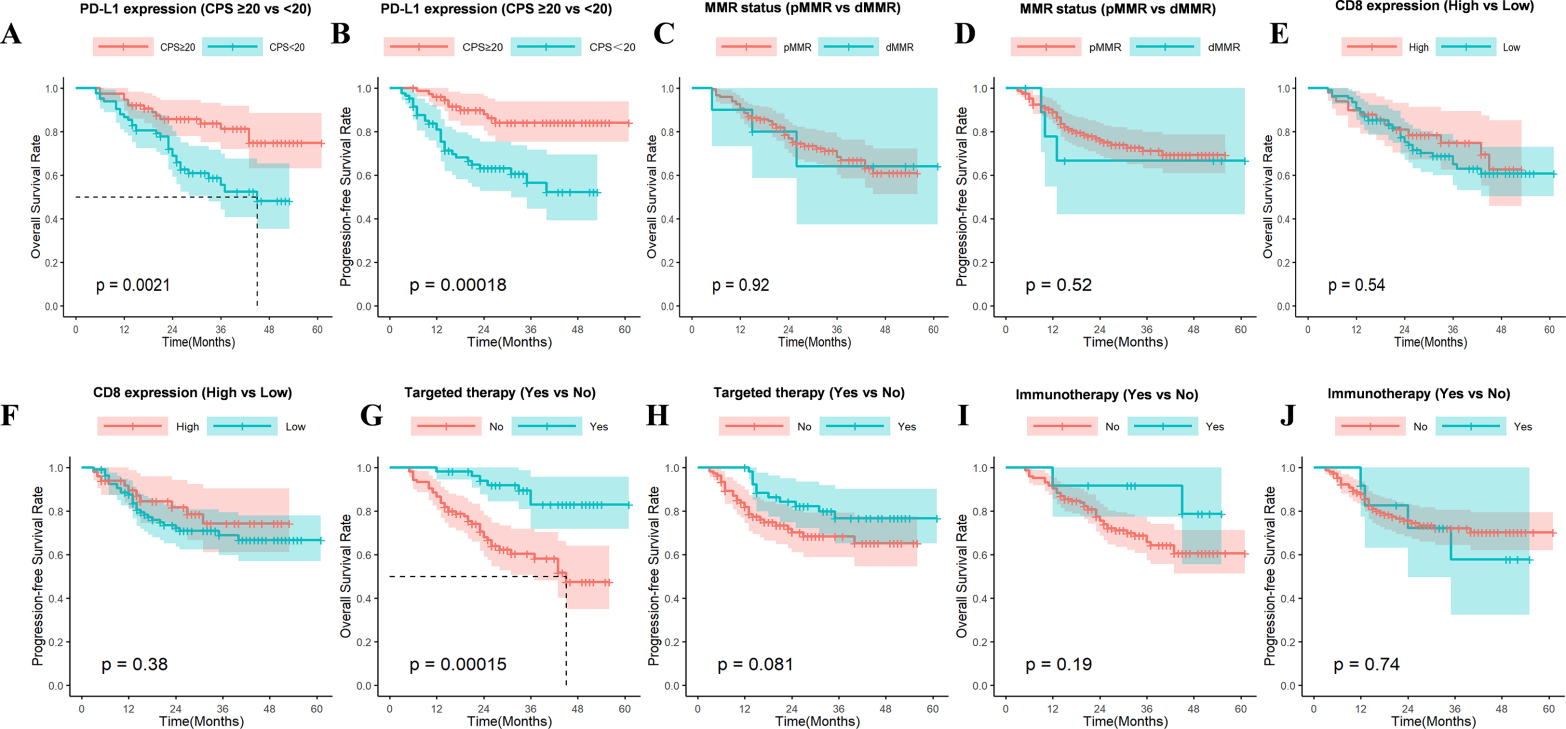
Kaplan–Meier analysis of OS and PFS. **(A, B)** High PD-L1 expression is significantly associated with better OS and PFS (OS, P = 0.0021; PFS, P = 0.00018); **(C, D)** MMR status is not related to patients’ OS and PFS (OS, P = 0.92; PFS, P = 0.52). **(E, F)** CD8 positive TILs show no statistically significant difference in OS and PFS (OS, P = 0.54; PFS, P = 0.38). **(G)** Patients with combined targeted therapy had significantly prolonged OS (P = 0.00015). **(H)** There is no statistical difference in PFS for patients with combined targeted therapy (P = 0.081). **(I, J)** Immunotherapy after chemoradiotherapy shows no significant benefit for OS and PFS in LA-HNSCC patients (OS, P = 0.19; PFS, P = 0.74). TILs, Tumor-Infiltrating Lymphocytes.

In this study, a total of 156 LA-HNSCC patients who had undergone chemoradiotherapy and had follow-up data were analyzed. Patients with high PD-L1 expression (CPS≥20) exhibited significantly improved overall survival (OS; P = 0.0021; **Figure 4A**) and progression-free survival (PFS; P<0.001; **Figure 4B**). Neither OS nor PFS differed significantly by MMR status (pMMR vs dMMR; P = 0.92 and P = 0.52, respectively; **Figures 4C, D**) or by CD8+ T-cell infiltration density (P = 0.54 for OS, P = 0.38 for PFS; **Figures 4E, F**). Receipt of targeted therapy was associated with a superior OS (P<0.001; **Figure 4G**) and a trend toward improved PFS that was not statistically significant (P = 0.081; **Figure 4H**). However, no significant survival benefit was observed in patients who received immunotherapy, for either OS (P = 0.19) or PFS (P = 0.74; **Figures 4I, J**).

### Univariate and multivariate analyses of prognostic factors in LA-HNSCC

**Figure 5:**
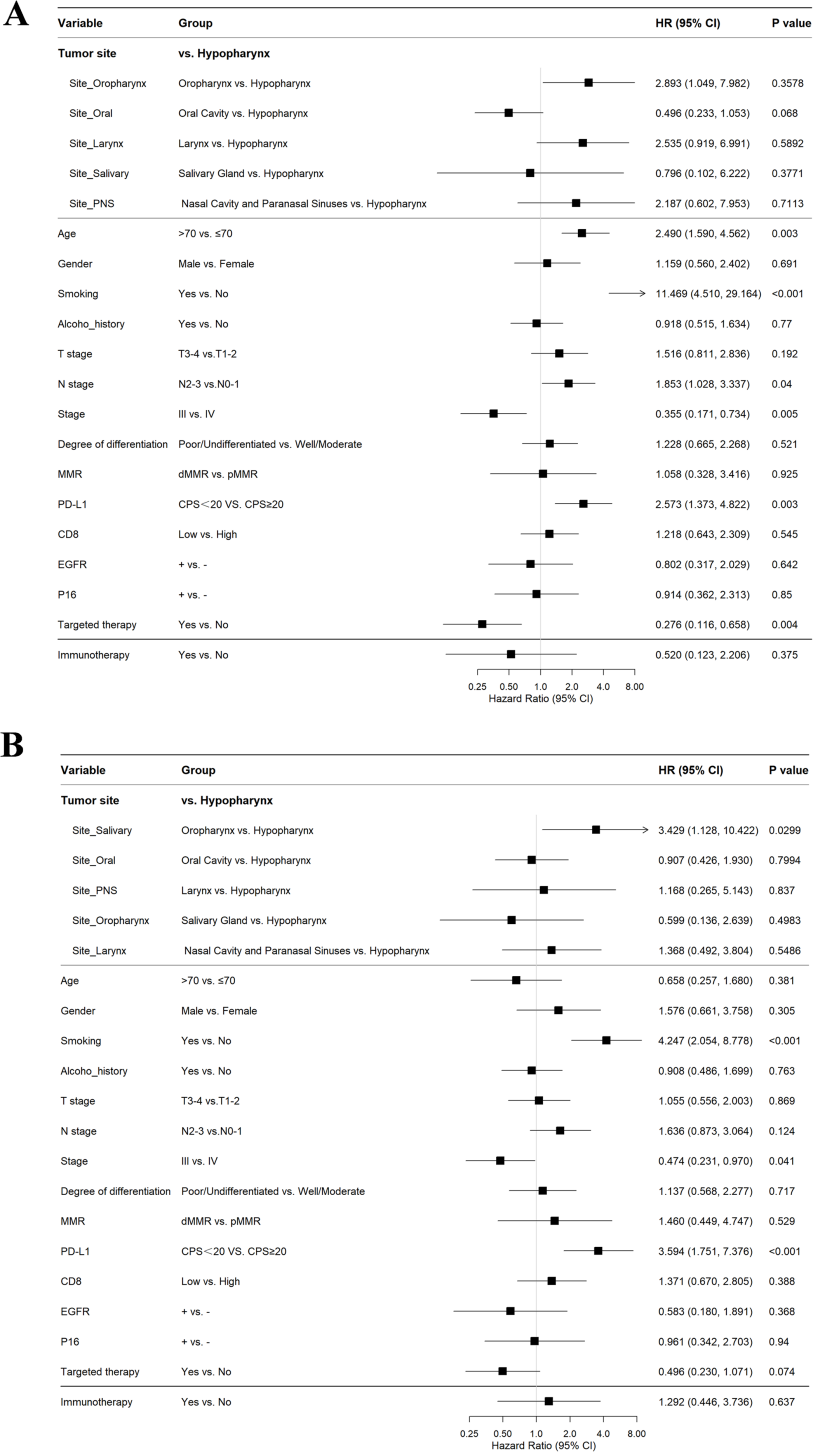
Univariate COX regression analysis. **(A)** age ≤ 70 years, history of smoking, stage N2-3, clinical stage IV, and high expression of PD-L1 (CPS≥20) are significant adverse prognostic factors for OS; **(B)** history of smoking, clinical stage IV, and high expression of PD-L1 (CPS≥20) are significant risk factors for disease progression.

**Figure 6:**
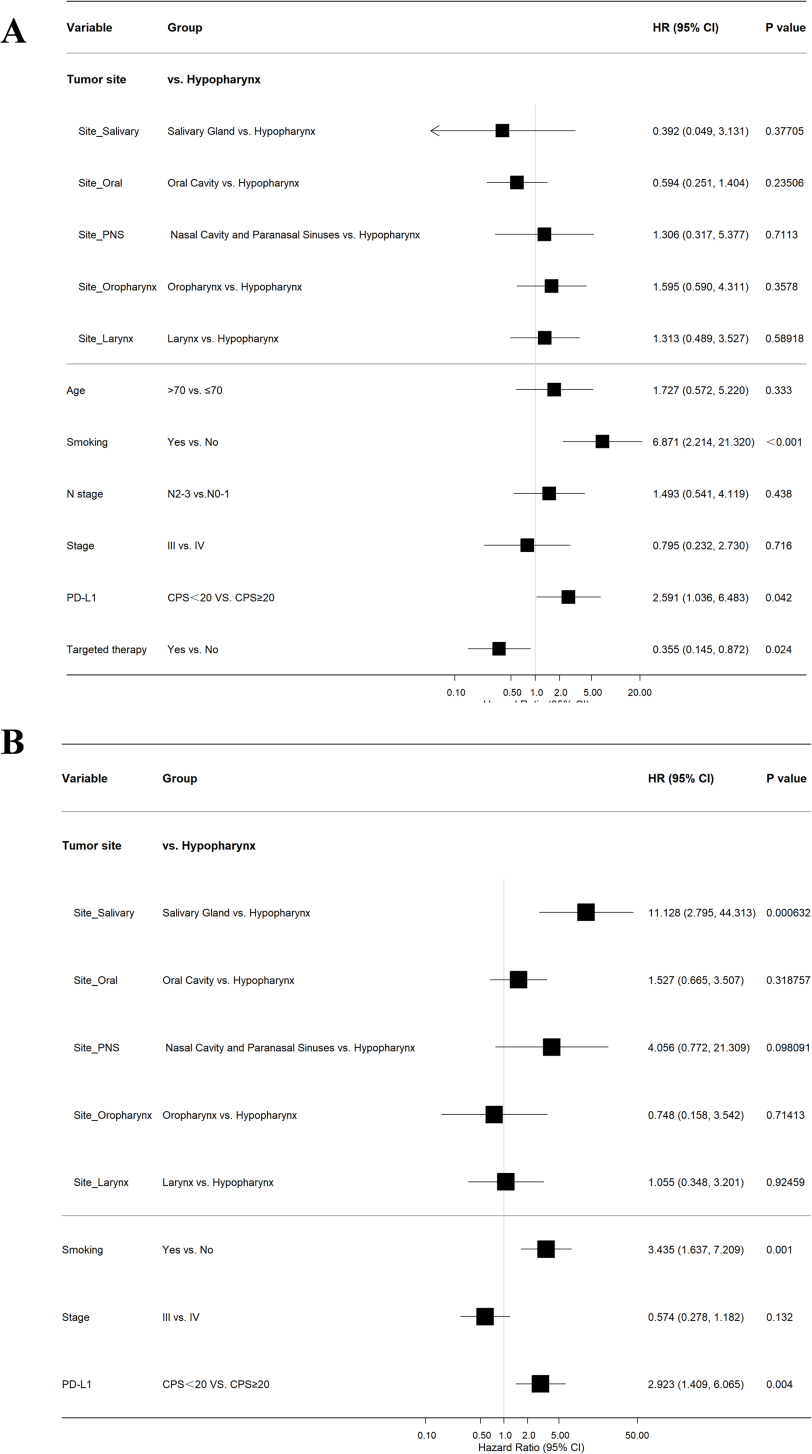
Multivariate COX regression analysis. **(A)** Smoking and high PD-L1 expression (CPS≥20) are independent risk factors for OS; **(B)** Smoking and high PD-L1 expression (CPS≥20) are independent risk factors for disease progression.

To identify independent prognostic factors for survival in patients with LA-HNSCC, univariate and multivariate Cox proportional hazards regression analyses were conducted for OS and PFS. Key results were visually summarized in **Figure 5** and **Figure 6**.

Univariate analysis for OS revealed several significant factors. Advanced age (>70 years; HR = 2.49, 95% CI: 1.59-4.56, P = 0.003), smoking history (non-smoker vs. smoker: HR = 11.47, 95% CI: 4.51-29.16, P<0.001), advanced nodal status (N2–3 vs. N0-1: HR = 1.85, 95% CI: 1.03-3.34, P = 0.04), and high PD-L1 expression (CPS≥20: HR = 2.57, 95% CI: 1.37-4.82, P = 0.003) were all associated with increased risk of mortality. Conversely, Stage IV disease (vs. Stage III: HR = 0.36, 95% CI: 0.17-0.73, P = 0.005) and administration of targeted therapy (no vs. yes: HR = 0.28, 95% CI: 0.12-0.66, P = 0.004) correlated with improved OS. All variables with P<0.05 were included in the multivariate model, which confirmed that smoking status (non-smoker vs. smoker: HR = 6.87, 95% CI: 2.21-21.32, P<0.001) and high PD-L1 expression (HR = 2.59, 95% CI: 1.04-6.48, P = 0.042) remained independent risk factors for worse OS. Targeted therapy persisted as an independent protective factor (HR = 0.36, 95% CI: 0.15-0.87, P = 0.024).

For PFS, univariate analysis indicated that smoking (non-smoker vs. smoker: HR = 4.25, 95% CI: 2.05-8.78, P<0.001) and high PD-L1 expression (CPS≥20: HR = 3.59, 95% CI: 1.75-7.38, P<0.001) were significant predictors of disease progression or death. Stage IV disease again correlated with improved PFS compared to Stage III (HR = 0.47, 95% CI: 0.23-0.97, P = 0.041). Multivariate analysis confirmed that both smoking (HR = 3.44, 95% CI: 1.64-7.21, P = 0.001) and PD-L1 expression (HR = 2.92, 95% CI: 1.41-6.07, P = 0.004) retained independence as adverse prognostic factors for PFS.

## Discussion

The primary objective of this study was to evaluate the predictive utility of PD-L1 expression and dMMR status in patients with LA-HNSCC undergoing CCRT. Our findings demonstrate that elevated PD-L1 expression (CPS≥20) serves as a robust predictor of improved therapeutic response and survival outcomes, including both OS and PFS. Specifically, within the cohort of 156 patients, those with high PD-L1 expression exhibited a treatment response rate of 97.1%, significantly surpassing that of the low-expression group. Multivariable analysis further established high PD-L1 expression as an independent factor associated with enhanced OS and PFS. Conversely, although dMMR status was associated with a lower response rate (50.0% vs. 79.5%), it unexpectedly did not translate into significant survival differences. Additionally, multivariate analysis confirmed that smoking status was an independent negative prognostic factor, whereas targeted therapies (such as anti-EGFR therapies) demonstrated clear survival benefits, further validating the established treatment paradigm.

The discovery of PD-L1 as a predictive biomarker holds significant clinical and biological importance. In the multivariate Cox analysis, after adjusting for other confounding factors such as age, stage, smoking status, and targeted therapy, high PD-L1 expression (CPS≥20) remained an independent predictor for both OS (HR = 2.591, 95% CI: 1.036-6.483, P = 0.042) and PFS (HR = 2.923, 95% CI:1.409-6.065, P = 0.004). Its association with high therapeutic response rates and survival benefits aligns with recent research findings, challenging the traditional view of PD-L1 as a marker of immune evasion (24, 25). We hypothesize that its potential mechanism may involve the synergistic effects of chemoradiotherapy and the tumor immune microenvironment. High PD-L1 expression often indicates a state of adaptive immune resistance, suggesting a pre-existing but suppressed T-cell immune response within the tumor microenvironment (26, 27). CCRT can induce immunogenic cell death, releasing a large number of tumor-associated antigens and danger signaling molecules, thereby further activating and amplifying pre-existing tumor-specific T-cell clones (28, 29). Therefore, high PD-L1-expressing “hot tumors” may be more sensitive to the antitumor immune effects triggered by CCRT, resulting in a synergistic effect. Our results showed that high PD-L1 expression was significantly associated with clinical characteristics such as primary oral tumors and non-smoking status, which often overlapped with HPV-negative (p16-negative) HNSCC. It was noteworthy that since p16 positivity did not show significant prognostic value in univariate analysis within our study cohort (P>0.05), and its value as a biomarker was mainly restricted to the oropharyngeal cancer subgroup, it was not included in the multivariate analysis model. This group typically exhibits a higher frequency of EGFR mutations and different tumor microenvironment features (30, 31). The strong predictive power of PD-L1 across the overall population suggests its broad applicability. Based on the unique immune microenvironment characteristics of HPV-negative tumors, we speculate that the predictive value of PD-L1 may be particularly prominent in this subgroup. This hypothesis awaits validation through subsequent studies evaluating HPV/p16 status separately in oropharyngeal and non-oropharyngeal cancers. If confirmed, it will provide an important basis for optimizing treatment strategies for the HPV-negative population.

The most thought-provoking finding of this study lay in the contradictory results presented by the dMMR status. The results of this study demonstrated that LA-HNSCC patients with dMMR had a significantly lower treatment response rate to CCRT compared to those with pMMR. The treatment response rate was 79.5% in the pMMR group versus 50.0% in the dMMR group, and this difference was statistically significant (P = 0.031). dMMR/MSI-H is a recognized biomarker for immune therapy sensitivity in colorectal and endometrial cancers (32, 33), but it was extremely rare in HNSCC (with an incidence rate of 6.4% in this study), and its role in CCRT had been rarely reported. We found that dMMR tumors had a significantly poorer initial response to CCRT, possibly due to their inherent genomic instability and the resultant intratumoral heterogeneity, which may select for treatment-resistant subclones (34, 35). However, despite this poor initial response, the long-term survival of dMMR patients was no different from that of pMMR patients. This dissociation between its value as a predictive biomarker for CCRT resistance and its lack of prognostic value for survival presents a paradox, which may be explained by two non-mutually exclusive hypotheses.

The first is the salvage effect of subsequent ICI therapy. It is well established that dMMR/MSI-H tumors exhibit exceptional sensitivity to ICI (36, 37). In our cohort, although frontline immunotherapy use did not differ between groups, we noted that some dMMR patients who failed CCRT subsequently received ICI. These patients likely attained profound disease control from later-line immunotherapy, thereby compensating for their initial poor response to CCRT and equilibrating long-term survival outcomes. While the small number of such patients precludes formal statistical validation, this represents a clinically plausible mechanism.

The second hypothesis pertains to the unique tumor-immune dynamics of dMMR cancers. The high tumor mutational burden and neoantigen load create a highly immunogenic microenvironment (17, 18). This continuous immune activation, even in the absence of radiological complete remission after CCRT, may suppress rapid tumor progression, inducing a state of long-term disease stability or ‘immune equilibrium’. This altered progression pattern could allow patients to achieve survival comparable to their pMMR counterparts without achieving a classic objective response.

Therefore, our data reframe the role of dMMR in LA-HNSCC. It appears to function primarily as a ‘treatment modality selection biomarker’. It predicts initial resistance to CCRT but simultaneously flags a tumor that is highly susceptible to subsequent immunotherapy and may follow a distinct clinical course due to its inherent immunogenicity. This complex, multi-stage role explains why it is a poor standalone prognostic marker in the context of modern, multi-modal therapy.

The study had several limitations. First, its retrospective design might have introduced selection bias. Second, the sample size for dMMR patients was small (n=10), and although statistically significant differences were observed, conclusions needed to be validated in studies with larger samples. Additionally, the lack of data on later-line treatments (particularly immunotherapy) prevented accurate assessment of their impact on survival outcomes for dMMR patients. Lastly, the study focused on clinical relevance analysis, and the proposed mechanistic hypotheses needed further validation through fundamental experiments, such as *in vivo* and *in vitro* models.

## Conclusion

In conclusion, this study confirmed that high PD-L1 expression was a positive predictive biomarker for CCRT benefit in patients with LA-HNSCC. Although dMMR status was associated with resistance to initial CCRT, its distinct immunogenic profile appears to influence subsequent disease progression, providing a strong rationale for selecting later-line therapies such as immunotherapy. These findings underscore the clinical value of MMR testing in LA-HNSCC, which primarily lies in identifying patients who are likely to be insensitive to initial CCRT but have a high probability of benefiting from subsequent immunotherapy, thereby informing personalized sequential treatment strategies.

## Data Availability

The raw data supporting the conclusions of this article will be made available by the authors, without undue reservation.

## Supplementary material

SUPPLEMENTARY FIGURE S1 Stratified analysis of objective response rate (ORR) by primary tumor site. Bar graph showing the ORR (percentage of patients achieving complete or partial response according to RECIST 1.1) among patients with LA-HNSCC treated with concurrent chemoradiotherapy, stratified by primary tumor subsite. Objective response rates were similar across all anatomical subsites. Statistical comparison using Chi-square test revealed no significant difference in ORR among the subgroups (P>0.05). This indicates that the initial treatment efficacy of CCRT was consistent irrespective of the primary tumor location in this cohort.

SUPPLEMENTARY FIGURE S2 Kaplan-Meier survival analysis by primary tumor site in patients with LA-HNSCC treated with concurrent chemoradiotherapy. **(A)** OS and **(B)** PFS stratified by the anatomical subsite of the primary tumor. Kaplan-Meier curves illustrate no significant difference in OS (P=0.85) or PFS (P=0.72) across different primary sites, indicating that tumor location did not independently influence long-term survival outcomes in this cohort. LA-HNSCC, locally advanced head and neck squamous cell carcinoma.

SUPPLEMENTARY FIGURE S3 Representative imaging of LA-HNSCC patients before and after CCRT. **(A, B)** Axial contrast-enhanced CT images of a 69-year-old male with hypopharyngeal carcinoma, obtained before and after treatment. **(A)** Baseline image shows circumferential wall thickening of the hypopharynx (red arrow). **(B)** Follow-up image at 3 months after CCRT completion demonstrates complete resolution of the primary tumor lesion (red arrow), assessed as CR. **(C, D)** Axial contrast-enhanced CT images of an 80-year-old male with hypopharyngeal carcinoma, before and after treatment. **(C)** Baseline image shows an enlarged lymph node in the left neck (red arrow). **(D)** Follow-up image at 3 months after CCRT completion reveals significant shrinkage of the lymph node (red arrow), assessed as PR. **(E, F)** Axial contrast-enhanced CT images of a 76-year-old male with gingival cancer before and after treatment. **(E)** Baseline image shows an irregular soft tissue mass in the right gingival region (red arrow). **(F)** Follow-up image at 3 months after CCRT completion indicates that the lesion did not shrink sufficiently to meet the criteria for partial response (red arrow), assessed as SD.

SUPPLEMENTARY FIGURE S4 Sensitivity analysis under a worst-case scenario for patients lost to follow-up. Kaplan–Meier survival curves illustrating OS and PFS after incorporating the worst-case assumption that all 9 patients lost to follow-up experienced immediate death (for OS) or disease progression (for PFS). **(A)** OS by PD-L1 expression (CPS≥20 vs. <20): high PD-L1 expression remained significantly associated with better OS (P=0.0093). **(B)** PFS by PD-L1 expression: high PD-L1 expression remained significantly associated with better PFS (P=0.00097). **(C)** OS by MMR status (pMMR vs. dMMR): no significant difference between groups (P=0.91). **(D)** PFS by MMR status: no significant difference between groups (P=0.67).
