# Supplementary material for: PD-L1 expression and mismatch repair deficiency in locally advanced head and neck squamous cell carcinoma treated with chemoradiotherapy: association with treatment response and survival
Source: Front Immunol. 2026 Jan 12;16:1709512. doi: 10.3389/fimmu.2025.1709512 (PMC12832915; doi:10.3389/fimmu.2025.1709512)
Supplement: Supplementary file 1 [file DataSheet1.docx]

**Table S1. Comparison of Baseline Characteristics Between Patients Lost to Follow-up and Those Finally Included**

| Characteristic | Lost to follow-up | Finally included | P-value |
| --- | --- | --- | --- |
| Age |  |  | 0.074 |
| ≤70 | 5（55.6%） | 123(78.8%) |  |
| >70 | 4（44.4%） | 33(21.2%) |  |
| Gender |  |  | 0.706 |
| Female | 1（11.1%） | 31(19.9%) |  |
| Male | 8（88.9%） | 125(80.1%) |  |
| Smoking history |  |  | 0.183 |
| No | 2（28.6%） | 74(47.4%) |  |
| Yes | 7（71.4%） | 82(52.6%) |  |
| Alcohol history |  |  | 0.090 |
| No | 1（11.1%） | 64(41.0%) |  |
| Yes | 8（88.9%） | 92(59.0%) |  |
| Tumor site |  |  | 0.717 |
| Salivary glands | 0 | 6(3.8%) |  |
| Oral cavity | 1（11.1%） | 54(34.6%) |  |
| Nasal sinuses | 0 | 7(4.5%) |  |
| Oropharynx | 0 | 15(9.6%) |  |
| Larynx | 2（28.6%） | 16(10.3%) |  |
| Hypopharynx | 6（66.7%） | 58(37.2%) |  |
| T-stage |  |  | 0.183 |
| T1-2 | 6（66.7%） | 58(37.2%) |  |
| T3-4 | 3（33.3%） | 98(62.8%) |  |
| N-stage |  |  | 0.517 |
| N0-1 | 3（33.3%） | 80(51.3%) |  |
| N2-3 | 6（66.7%） | 76(48.7%) |  |
| Stage |  |  | 0.852 |
| III | 2（28.6%） | 57(36.5%) |  |
| IV | 7（71.4%） | 99(63.5%) |  |
| Grade |  |  | 0.178 |
| Well/Moderate | 6（66.7%） | 114(73.1%) |  |
| Poor/Undifferentiated | 3（33.3%） | 42(26.9%) |  |
| PD-L1 |  |  | 0.337 |
| CPS>20 | 3（33.3%） | 74(47.4%) |  |
| CPS≤20 | 6（66.7%） | 82(52.6%) |  |
| MMR |  |  | ＞0.999 |
| pMMR | 9（100%） | 146(93.6%) |  |
| dMMR | 0 | 10(6.4%) |  |
| CD8 |  |  | 0.651 |
| High | 3（33.3%） | 49(31.4%) |  |
| Low | 6（66.7%） | 107(68.6%) |  |
| EGFR |  |  | 0.693 |
| + | 8（88.9%） | 137(87.8%) |  |
| - | 1（11.1%） | 19(12.2%) |  |
| P16 |  |  | 0.615 |
| + | 0 | 17(10.9%) |  |
| - | 9（100%） | 139(89.1%) |  |
| Targeted therapy |  |  | 0.517 |
| No | 7（71.4%） | 104(66.7%) |  |
| Yes | 2（28.6%） | 52(33.3%) |  |
| Immunotherapy |  |  | 0.606 |
| No | 9（100%） | 144(92.3%) |  |
| Yes | 0 | 12(7.7%) |  |
