# Supplementary figures and images for: PD-L1 expression and mismatch repair deficiency in locally advanced head and neck squamous cell carcinoma treated with chemoradiotherapy: association with treatment response and survival
Source: Front Immunol. 2026 Jan 12;16:1709512. doi: 10.3389/fimmu.2025.1709512 (PMC12832915; doi:10.3389/fimmu.2025.1709512)

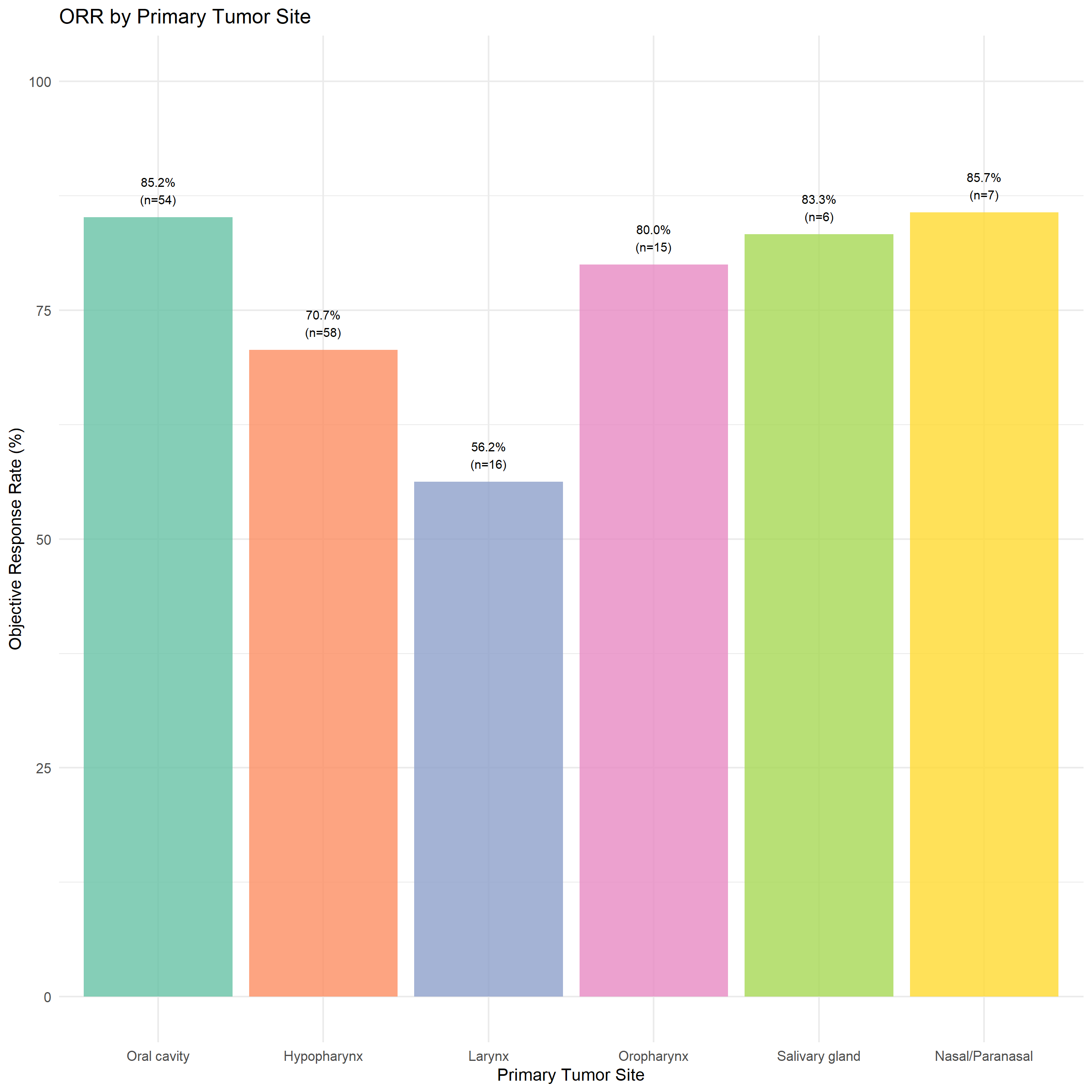

Supplement: SUPPLEMENTARY FIGURE S1 — Stratified analysis of objective response rate (ORR) by primary tumor site. Bar graph showing the ORR (percentage of patients achieving complete or partial response according to RECIST 1.1) among patients with LA-HNSCC treated with concurrent chemoradiotherapy, stratified by primary tumor subsite. Objective response rates were similar across all anatomical subsites. Statistical comparison using Chi-square test revealed no significant difference in ORR among the subgroups (P>0.05). This indicates that the initial treatment efficacy of CCRT was consistent irrespective of the primary tumor location in this cohort. [file Image1.tiff]

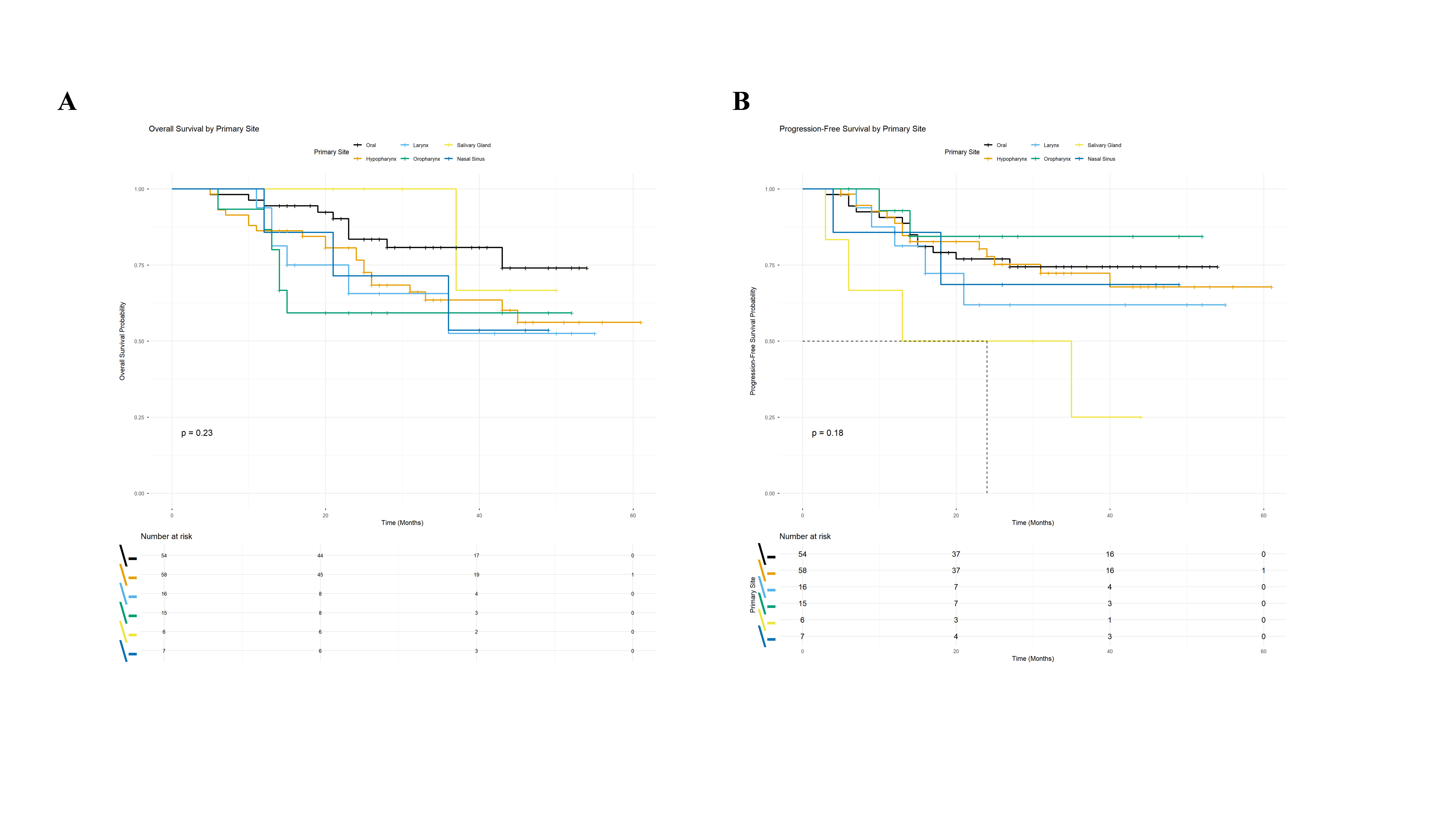

Supplement: SUPPLEMENTARY FIGURE S2 — Kaplan-Meier survival analysis by primary tumor site in patients with LA-HNSCC treated with concurrent chemoradiotherapy. (A) OS and (B) PFS stratified by the anatomical subsite of the primary tumor. Kaplan-Meier curves illustrate no significant difference in OS (P=0.85) or PFS (P=0.72) across different primary sites, indicating that tumor location did not independently influence long-term survival outcomes in this cohort. LA-HNSCC, locally advanced head and neck squamous cell carcinoma. [file Image2.tif]

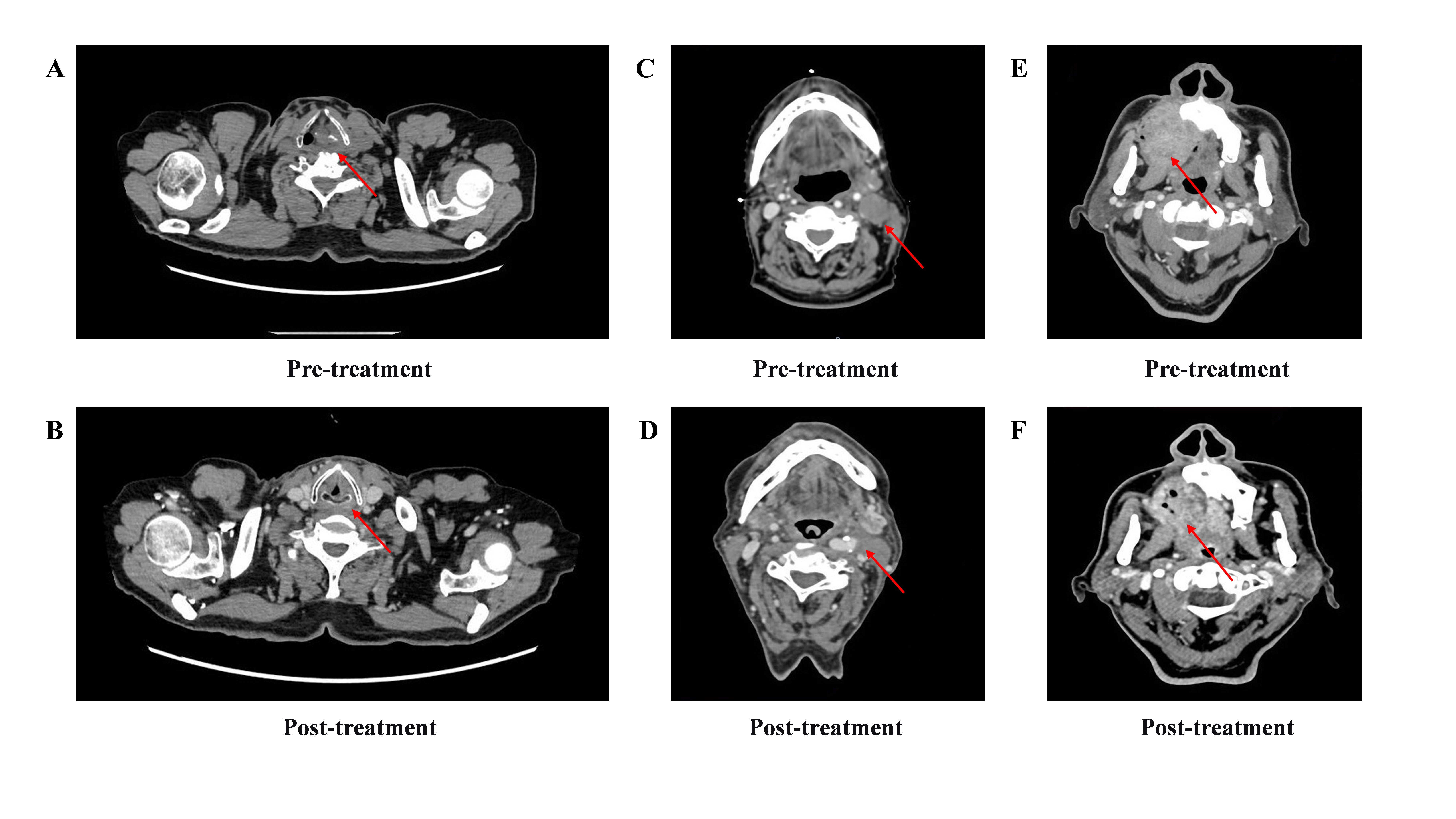

Supplement: SUPPLEMENTARY FIGURE S3 — Representative imaging of LA-HNSCC patients before and after CCRT. (A, B) Axial contrast-enhanced CT images of a 69-year-old male with hypopharyngeal carcinoma, obtained before and after treatment. (A) Baseline image shows circumferential wall thickening of the hypopharynx (red arrow). (B) Follow-up image at 3 months after CCRT completion demonstrates complete resolution of the primary tumor lesion (red arrow), assessed as CR. (C, D) Axial contrast-enhanced CT images of an 80-year-old male with hypopharyngeal carcinoma, before and after treatment. (C) Baseline image shows an enlarged lymph node in the left neck (red arrow). (D) Follow-up image at 3 months after CCRT completion reveals significant shrinkage of the lymph node (red arrow), assessed as PR. (E, F) Axial contrast-enhanced CT images of a 76-year-old male with gingival cancer before and after treatment. (E) Baseline image shows an irregular soft tissue mass in the right gingival region (red arrow). (F) Follow-up image at 3 months after CCRT completion indicates that the lesion did not shrink sufficiently to meet the criteria for partial response (red arrow), assessed as SD. [file Image3.tif]

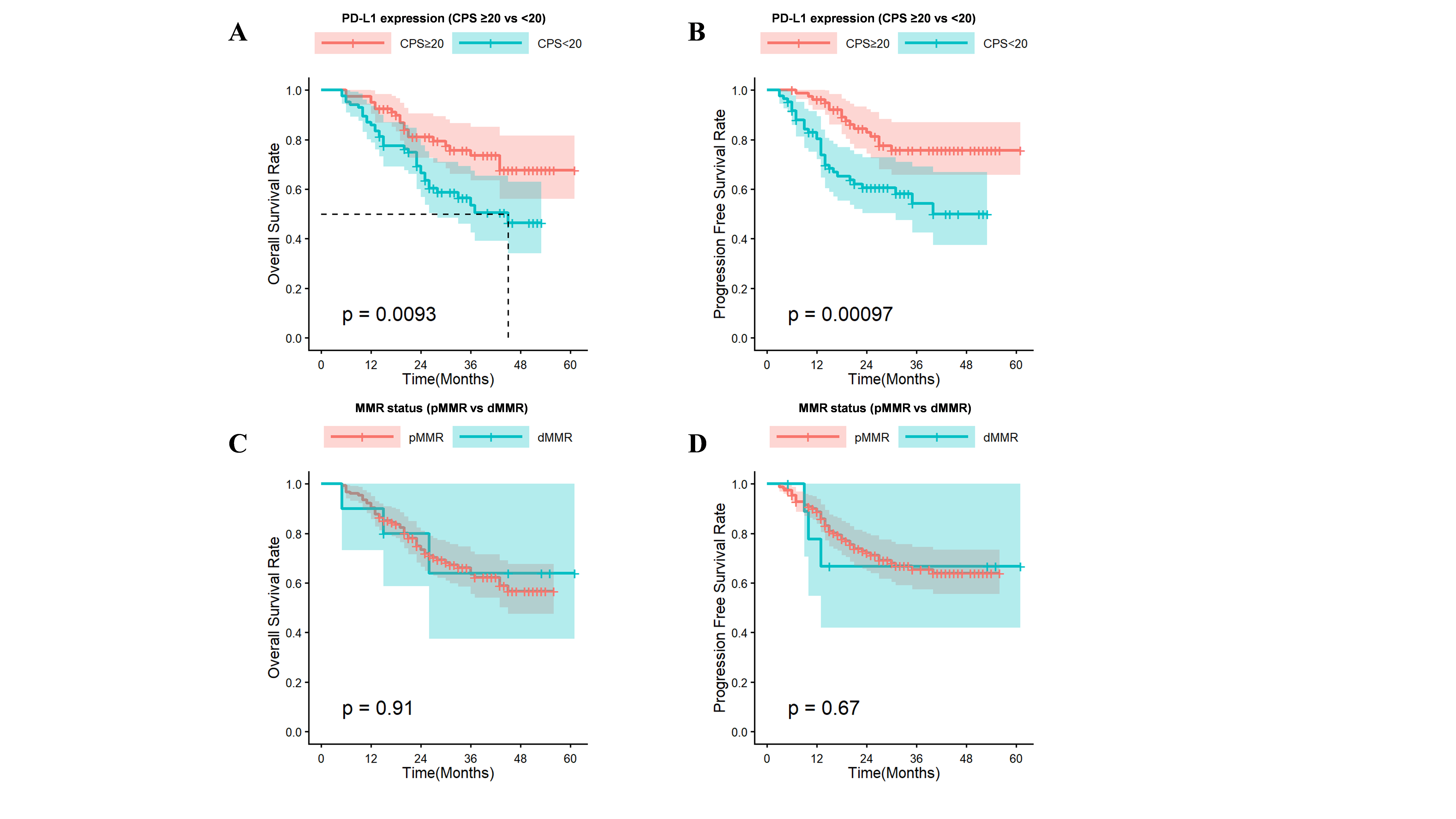

Supplement: SUPPLEMENTARY FIGURE S4 — Sensitivity analysis under a worst-case scenario for patients lost to follow-up. Kaplan–Meier survival curves illustrating OS and PFS after incorporating the worst-case assumption that all 9 patients lost to follow-up experienced immediate death (for OS) or disease progression (for PFS). (A) OS by PD-L1 expression (CPS≥20 vs. <20): high PD-L1 expression remained significantly associated with better OS (P=0.0093). (B) PFS by PD-L1 expression: high PD-L1 expression remained significantly associated with better PFS (P=0.00097). (C) OS by MMR status (pMMR vs. dMMR): no significant difference between groups (P=0.91). (D) PFS by MMR status: no significant difference between groups (P=0.67). [file Image4.tif]
